# Removing rician bias in diffusional kurtosis of the prostate using real‐data reconstruction

**DOI:** 10.1002/mrm.28080

**Published:** 2019-11-18

**Authors:** Rosie J. Goodburn, Tristan Barrett, Ilse Patterson, Ferdia A. Gallagher, Edward M. Lawrence, Vincent J. Gnanapragasam, Christof Kastner, Andrew N. Priest

**Affiliations:** ^1^ Department of Medical Physics Cambridge University Hospitals NHS Foundation Trust Cambridge United Kingdom; ^2^ Division of Radiotherapy and Imaging The Institute of Cancer Research London; ^3^ Department of Radiology School of Clinical Medicine University of Cambridge Cambridge United Kingdom; ^4^ Department of Radiology Cambridge University Hospitals NHS Foundation Trust Cambridge United Kingdom; ^5^ Academic Urology Group Department of Surgery University of Cambridge Cambridge United Kingdom; ^6^ Department of Urology Cambridge University Hospitals NHS Foundation Trust Cambridge United Kingdom

**Keywords:** diffusion weighted MRI, diffusional kurtosis MRI, prostate cancer, quantitative imaging

## Abstract

**Purpose:**

To compare prostate diffusional kurtosis imaging (DKI) metrics generated using phase‐corrected real data with those generated using magnitude data with and without noise compensation (NC).

**Methods:**

Diffusion‐weighted images were acquired at 3T in 16 prostate cancer patients, measuring 6 *b*‐values (0‐1500 s/mm^2^), each acquired with 6 signal averages along 3 diffusion directions, with noise‐only images acquired to allow NC. In addition to conventional magnitude averaging, phase‐corrected real data were averaged in an attempt to reduce rician noise‐bias, with a range of phase‐correction low‐pass filter (LPF) sizes (8‐128 pixels) tested. Each method was also tested using simulations. Pixelwise maps of apparent diffusion (*D*) and apparent kurtosis (*K*) were calculated for magnitude data with and without NC and phase‐corrected real data. Average values were compared in tumor, normal transition zone (NTZ), and normal peripheral zone (NPZ).

**Results:**

Simulations indicated LPF size can strongly affect *K* metrics, where 64‐pixel LPFs produced accurate metrics. Relative to metrics estimated from magnitude data without NC, median NC *K* were lower (*P* < 0.0001) by 6/11/8% in tumor/NPZ/NTZ, 64‐LPF real‐data *K* were lower (*P* < 0.0001) by 4/10/7%, respectively.

**Conclusion:**

Compared with magnitude data with NC, phase‐corrected real data can produce similar *K*, although the choice of phase‐correction LPF should be chosen carefully.

## 1 INTRODUCTION

Prostate cancer (PCa) is the most common cancer in men in developed countries.^1^ However, PCa encompasses a spectrum of low‐ to high‐risk diseases, which are often poorly characterized by current diagnostic methods, leading to both overtreatment and undertreatment.^2^ Diffusion‐weighted imaging (DWI), together with one of its metrics, the apparent diffusion coefficient (ADC), has shown promise as a noninvasive tool for improved PCa risk stratification,^3^ although significant overlap still exists between ADC values for high‐ and low‐grade tumors.^4^, ^5^, ^6^, ^7^, ^8^, ^9^, ^10^

Standard DWI is quantified using a monoexponential model of signal decay with *b*‐value, which is derived from the simplified assumption of Gaussian diffusion, where water diffuses freely according to Brownian motion. However, water diffusion in biological tissue is much more complex and often deviates substantially from Gaussian behavior.^11^ Consequently, several “non‐Gaussian” signal models have been suggested to more accurately describe in vivo diffusion.^12^, ^13^, ^14^, ^15^, ^16^, ^17^, ^18^, ^19^, ^20^, ^21^, ^22^ One such expanded model is diffusional kurtosis imaging (DKI),^12^ which provides an estimate of the apparent diffusional kurtosis (*K*): a dimensionless parameter that quantifies the degree of non‐Gaussian diffusion. Since the first DKI studies,^12^, ^23^ the technique has been applied extensively in neuroimaging^24^, ^25^, ^26^ and other studies have demonstrated feasibility for body applications, including PCa.^27^, ^28^, ^29^, ^30^, ^31^, ^32^, ^33^, ^34^ Several investigations have compared DKI with standard DWI in PCa,^29^, ^30^, ^31^, ^32^, ^33^, ^34^ with disagreement over whether DKI brings additional value compared to standard DWI^29^, ^30^, ^31^ or not.^32^, ^33^, ^34^ However, these findings may be influenced by methodological issues, including the choice of *b*‐values and by whether and how noise compensation (NC) is performed, and this issue has yet to be fully investigated.

DKI acquisition protocols use DWI pulse sequences with a range of low to ultrahigh *b*‐values, required to measure *K* accurately.^35^ Unfortunately, higher *b*‐values are associated with low signal‐to‐noise ratios (SNRs). In such cases, the rician noise distribution in magnitude‐reconstructed data introduces an increased mean pixel value,^36^ called the “rectified noise floor,”^37^ where the effect of this on the fitted DKI model is to mimic the presence of diffusional kurtosis, causing an upward bias in *K*.

Most low‐SNR MRI applications are improved by use of complex signal‐averaging to increase SNR and, hence, reduce noise‐floor bias, although complex averaging is generally avoided in DWI, where motion‐induced phase shifts lead to destructive interference between averaged data and severe loss of signal.^36^ In body DWI, each *b*‐value image is typically calculated from 12‐24 signal averages (3 diffusion directions and 4‐8 signal excitations [NeX]), applying the magnitude operation before averaging to avoid phase‐interference artifacts. However, while magnitude averaging does reduce apparent noise, it does not reduce the upward bias of the noise floor.^36^, ^37^

Several methods have been proposed to remove noise‐floor bias from DWI.^36^, ^37^, ^38^, ^39^, ^40^, ^41^, ^42^, ^43^ A common approach is to use a correction‐scheme to compensate for biasing effects in noisy magnitude images.^37^, ^38^, ^39^, ^40^, ^41^ However, these techniques require knowledge of the noise statistics in a specific region of interest (ROI) and this is not easily determined in body DWI, especially on a voxelwise basis.^36^ Alternatively, NC methods see rician statistics taken into account in fitting the diffusion model,^42^ although this requires the acquisition of additional noise‐only images at the expense of longer scanning times. A third approach is to reconstruct phase‐corrected real data in an attempt to maintain the original noise statistics with no rectified noise floor.^36^, ^37^, ^43^ The purpose of this study is to investigate the effects of phase‐corrected real data for DKI in the prostate compared to standard magnitude data, with and without NC.

## 2 METHODS

All data analysis was carried out in MATLAB (Mathworks, Natick, MA, USA).

### 2.1 Patients

Sixteen patients with clinical suspicion of undiagnosed PCa were prospectively enrolled into this local institutional review board‐approved (CUH/13/EE/0100) single‐center study, with all subjects signing written informed consent. Inclusion criteria were either prior negative biopsy or biopsy‐proven low risk PCa and an MRI suspicious for a new high‐grade tumor. This patient group was a subset of that used for a previous study.^28^

### 2.2 MRI

All patients underwent 3T MRI (Discovery MR750, GE‐Healthcare, WI, USA) using a 32‐channel phased‐array coil. A diffusion‐weighted (Stejskal‐Tanner dual‐spin‐echo EPI) sequence was used to acquire axial slices, with imaging parameters: echo time 95 ms; repetition time 6000 ms; 6 NeX; field of view (FOV) 28 × 28 cm^2^; slice thickness 3.6 mm; slice gap 0.4 mm; acquisition matrix 128 × 96; reconstruction matrix 256 × 256; parallel imaging (ASSET, essentially equivalent to the SENSE method)^44^ acceleration factor 2; 6 *b*‐values: 0, 100, 450, 800, 1150, 1500 s/mm^2^, with each non‐zero *b*‐value acquired along 3 orthogonal diffusion directions. For the purpose of NC, additional noise‐only images were collected using an identical acquisition and reconstruction but without radiofrequency excitation pulses.^45^ The raw (k‐space) data from both the DWI and ASSET calibration scans were stored to allow subsequent re‐analysis.

### 2.3 Data reconstruction

The manufacturer's research software (Orchestra SDK 1.6, GE Healthcare) was used to investigate and adapt DWI reconstruction. The relevant portion of the magnitude reconstruction function performs the following steps for each unique slice location, *b*‐value, diffusion‐direction, and NeX:

1. For each channel image, a high‐pass, preweighted image and a low‐pass image are calculated as part of homodyne detection,^46^ where preweighting corrects for partial sampling of k‐space and low‐pass images are used for phase correction.
2. Parallel imaging (ASSET) unaliasing is performed for both the preweighted and low‐pass channel images.
3. Homodyne phase correction is performed using the ASSET‐unaliased, preweighted, and low‐pass images, and the real part of this homodyne‐processed image is taken.
4. The magnitude of the real‐valued, homodyne‐processed image is taken.

These operations are repeated for each NeX, and the NeX images are averaged to give a magnitude‐averaged image.

This algorithm was adapted to produce a reconstruction method that generates phase‐corrected, real‐valued images. First, step 4 was removed to avoid the magnitude operation. Second, phase correction (which is already part of homodyne processing) was adapted so that only the very slowly varying phase differences were removed while preserving higher frequency phase variations assumed to be due to random noise. Since some functions provided by the manufacturer were “black‐boxes,” we could not directly change the filter. Instead, additional low‐pass filtering was performed using a 2D Fourier‐domain Hamming window on the ASSET‐unaliased, low‐pass image following step 2 to produce a “lower‐pass image,” used to perform phase correction:

1. For each channel image, a high‐pass, preweighted image and a low‐pass image are calculated as part of homodyne detection.
2. Parallel imaging (ASSET) unaliasing is performed for both the preweighted and low‐pass channel images.
3. A lower‐pass image is calculated from the ASSET‐unaliased, low‐pass image via a 2D Fourier‐domain Hamming window.
4. Homodyne phase correction is performed using the ASSET‐unaliased, preweighted and lower‐pass images, and the real part of this homodyne‐processed image is taken.

The optimal size of the Hamming window was determined via the use of digital‐phantom simulations described in the next section. The resulting real‐valued images were accumulated and averaged in the same way as for the original reconstruction.

### 2.4 Simulations

To investigate optimal low‐pass filter (LPF) sizes, we tested a simple real‐data reconstruction method with phase correction using a range of LPF sizes for a digital phantom with simulated noise. The phantom was built as a 2D complex image (256 × 256) with independent, random Gaussian noise added to the real and imaginary parts. To simulate the effect of motion, the image phase (wrapped onto the range of [−π, π]) was offset by a random number between −π and π (translation) and scaled by a random slope within a range that varied with *b*‐value, based on inspection of clinical data (Supporting Information Figure S1, which is available online). The reconstruction method described above (without ASSET‐unaliasing) was applied to a fully‐sampled, full‐Fourier k‐space, except that parallel imaging was not included. To mimic the preweighting filter, images were low‐pass filtered with a 200‐pixel Hamming window, (chosen to produce visually similar phase when compared with clinical data). To reflect the patient protocol, 18 (6 NeX, 3 diffusion directions) repetitions were generated. Both magnitude‐averaged and phase‐corrected, real‐averaged images were constructed.

**Figure 1:**
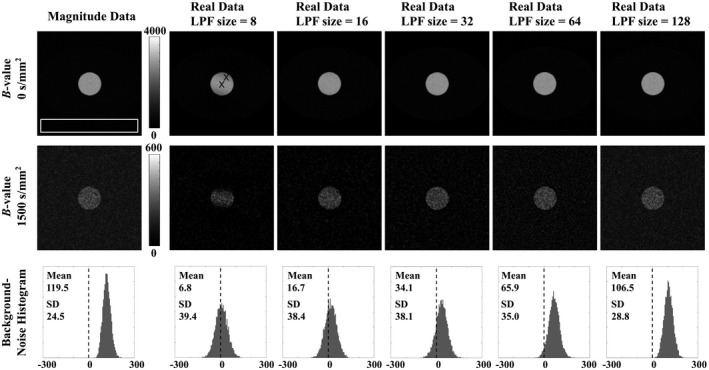
6‐NeX phantom images and background‐region (white box) noise histograms for magnitude‐ and real‐data reconstructions. Real‐data phase‐correction was performed with LPF sizes of 8, 16, 32, 64, and 128 pixels. Shown for a true signal of 2800 (*b* = 0 smm^−2^) and 233 (*b* = 1500 smm^−2^) in the high‐signal region and a SD of noise equal to 310 for each NeX, where central and off‐center crosses indicate where pixels may yield different results due to the effect of artifacts

For the real‐averaged images, several sizes of 2D, low‐pass Hamming‐window filters were tested, ranging 8 to 128 pixels. Figure 1 illustrates 2 effects that appear to be influenced by the size of the LPF: smaller filter sizes generate more severe phase artifacts, while larger filter sizes produce positive‐mean noise distributions, as observed in image histograms. Phase‐cancellation artifacts, manifesting as regions of low signal intensity, are here especially apparent in the high‐*b*‐value image for the real‐data phased‐corrected with an 8‐pixel LPF.

For each simulated dataset, images were generated using 6 signal values from *S*(0) = 1500/2800, based on clinical values in tumor/normal tissue, over 6 *b*‐values from 0 to 1500 s/mm^2^ using simulated tumor/normal tissue apparent diffusion (*D*) and *K* values of 1.25/2.31 × 10^−3^ mm^2^/s and 0.83/0.48, respectively (Supporting Information Table S1). All “raw” data was confounded with noise SD of 310.

To reflect the patient measurements (see the Diffusional kurtosis model fitting section), each of the 3 NeX groups (diffusion directions) were fitted independently using the diffusional kurtosis model (Equation 1); *D* and *K* were calculated as the arithmetic mean of the fitted values. Fits were performed for 2 representative pixels; a pixel in the center and off‐center of the high‐signal region that were differently affected by phase‐cancellation artifact (marked by Xs in Figure 1). This process was repeated for 1000 magnitude‐ and real‐data simulated reconstructions of the digital phantom.

### 2.5 Diffusional kurtosis model fitting

Pixelwise nonlinear fits of the magnitude‐ and real‐data patient datasets were performed independently for each diffusion direction using:

(1) $$S\left(b\right)=S\left(0\right)\;\exp\left(-bD\;+\;b^{2}D^{2}K/6\right)$$

to generate maps of *D* and *K* with fitting constraints^35^:

- *D* (×10^−3^ mm^2^/s): lower bound 0.001; upper bound 5.
- *DK* (×10^−3^ mm^2^/s): lower bound 0; upper bound 3/*b*_max_.

Our in‐house fitting method was written in Matlab (Mathworks, Natick, MA, USA) to fit data using the Trust‐Region Reflective algorithm. The code included an NC option to compensate for rectified noise floor in magnitude data, for which the model fitted was an estimate of the measured noisy signal, *S_n_*, given by:

(2) $$S_{n}=\sqrt{S^{2}\;+\;n^{2}}$$

where *S* is an estimate of the true signal, and the noise parameter, *n*, is estimated from the mean of the separately acquired noise‐only images, smoothed by a 25 × 25 pixelwise adaptive Wiener filter and multiplied by $\sqrt{2/\pi }$ to correct for the mean value of a rician distribution at very low SNR.^12^, ^45^

Average *D* and *K* maps were calculated as the pixelwise, arithmetic mean of *D* and *K* maps produced for each diffusion direction. In total, 7 sets of (average) *D* and *K* maps were generated for each patient, calculated from magnitude data without NC, magnitude data with NC, and phase‐corrected real‐data reconstructed with LPF sizes of 8, 16, 32, 64, and 128 pixels. *D* and *K* maps were masked according to low‐signal thresholding in the zero‐*b*‐value images and pixels with negative *K* values were set to zero.

### 2.6 ROI analysis

ROIs were outlined in the prostate by 1 author (fellowship‐trained uroradiologist with 7 years’ clinical prostate MR reporting experience), with reference to ADC maps and T_2_‐weighted images. Delineation was performed for tumor as the largest single tumor region, and for tissue that was normal‐appearing on standard imaging sequences and benign on sector biopsies in the peripheral zone (NPZ) and transition zone (NTZ). These ROI volumes were then used to extract mean *D* and *K* in tumor, NPZ and NTZ.

### 2.7 Statistical analysis

Friedman tests were performed to compare mean *D* and *K* ROI metrics estimated using real data, magnitude data, and magnitude data with NC. Due to the sample size of the study, the paired Wilcoxon signed‐rank test was then used to compare these metrics. *P*‐values less than 0.05 were taken as significant.

## 3 RESULTS

### 3.1 Patients

Sixteen patients were scanned successfully. Their median age was 67 years (range, 50‐76 years), while the median prostate specific antigen level was 7.66 ng/mL (range, 0.74‐8.80 ng/mL).

### 3.2 Filter size tests

Table 1 summarizes the median and interquartile range (IQR) of the simulated DKI metrics. For models based on NPZ‐tissue *D* (2.31 × 10^−3^ mm^2^/s) and *K* (0.49) values, real data produces less biased *D* and *K* with LPF sizes of 32 and 64 pixels compared to magnitude data both with and without NC. However, for tumor‐based values, only 128‐pixel LPFs reduce overall biases, for which magnitude data without correction generates relatively accurate results. The use of NC appears to over‐correct *K* here, where this is improved upon by real data with LPF sizes of 64 and 128 pixels.

**Table 1:** Median (IQR) *D* and *K* values fitted from 1000 magnitude‐data, without and with NC, and real‐data digital‐phantom reconstruction simulations^a^

Data type	Apparent diffusion (D, x 10^−^^3^mm^2^/s)			Apparent kurtosis (K, unitless)		
	Central ROI	Off‐center ROI	Av. bias	Central ROI	Off‐center ROI	Av. bias
Noiseless sig.	2.31		‐	0.49		‐
Magnitude	2.33 (0.09)	2.33 (0.09)	0.7%	0.52 (0.06)	0.53 (0.06)	7.1%
Mag. & NC	2.31 (0.09)	2.31 (0.09)	0.2%	0.49 (0.07)	0.48 (0.07)	1.0%
Real, LPF8	2.31 (0.09)	2.81 (0.17)	10.9%	0.48 (0.07)	0.41 (0.10)	8.9%
Real, LPF16	2.31 (0.09)	2.37 (0.10)	1.5%	0.48 (0.07)	0.48 (0.07)	2.1%
Real, LPF32	2.32 (0.09)	2.31 (0.09)	0.2%	0.49 (0.08)	0.48 (0.07)	0.6%
Real, LPF64	2.31 (0.09)	2.31 (0.09)	0.2%	0.49 (0.07)	0.49 (0.07)	0.5%
Real, LPF128	2.32 (0.09)	2.33 (0.09)	0.6%	0.51 (0.07)	0.51 (0.06)	4.4%
Noiseless sig.	1.25		‐	0.83		‐
Magnitude	1.25 (0.10)	1.25 (0.10)	0.2%	0.85 (0.20)	0.84 (0.21)	1.6%
Mag. & NC	1.25 (0.10)	1.25 (0.10)	0.3%	0.80 (0.21)	0.79 (0.22)	4.3%
Real, LPF8	1.26 (0.10)	1.62 (0.16)	15.2%	0.79 (0.21)	0.46 (0.24)	24.8%
Real, LPF16	1.25 (0.11)	1.29 (0.11)	1.8%	0.79 (0.24)	0.75 (0.22)	7.5%
Real, LPF32	1.25 (0.10)	1.24 (0.10)	0.3%	0.78 (0.23)	0.78 (0.23)	5.9%
Real, LPF64	1.25 (0.10)	1.25 (0.10)	0.2%	0.80 (0.22)	0.80 (0.22)	3.7%
Real, LPF128	1.25 (0.11)	1.25 (0.11)	<0.1%	0.82 (0.22)	0.83 (0.21)	0.7%

a Average biases indicate the average absolute percentage differences from true DKI metrics for 2 central and off‐center (Figure 1) pixels. The 2 pairs of values correspond to NPZ (top) and tumor (bottom).

Considering the 2 models of signal decay, the 64‐pixel LPF produces the lowest total “average bias” for the pixels in artifactual and nonartifactual regions, based on the absolute percentage differences from the true *D* and *K* values. Although this filter size is not expected to fully preserve zero‐mean noise statistics, these simulations suggest that lower filter sizes introduce inaccuracies likely due to the effect of phase‐cancellation artifacts.

### 3.3 Magnitude and real images

**Figure 2:**
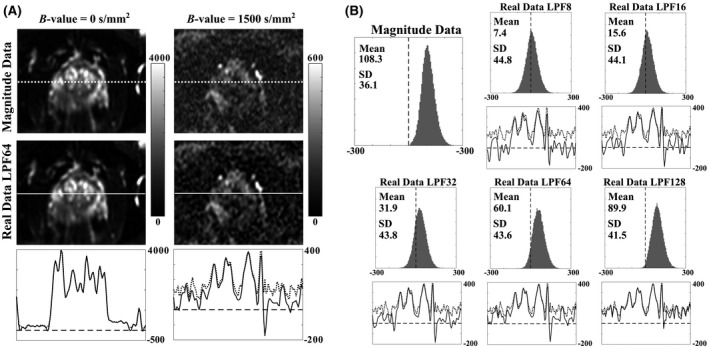
A, Same‐slice, 6 NeX magnitude‐ and real‐data (phase correction performed with an LPF size of 64 pixels) prostate images shown for *b*‐values of 0 and 1500 s/mm^2^ (right‐left diffusion direction). Images were cropped by 70‐90 pixels from each FOV edge. Bottom plots show intensity profiles of magnitude (dotted line) and real (solid line) data along the profiles in above images, where the dashed lines mark zero. For the high *b*‐value images, real data increases contrast for small intensity differences. B, Noise histograms of magnitude and real data (with phase correction performed with LPF sizes of 8, 16, 32, 64, and 128 pixels, respectively) from same‐slice noise images (6 NeX, single diffusion‐direction), where the true SNR is expected to be close to zero (dashed line). Also shown are intensity profiles of magnitude (dotted line) and real (solid line) data equivalent to those in Figure 2A

**Figure 3:**
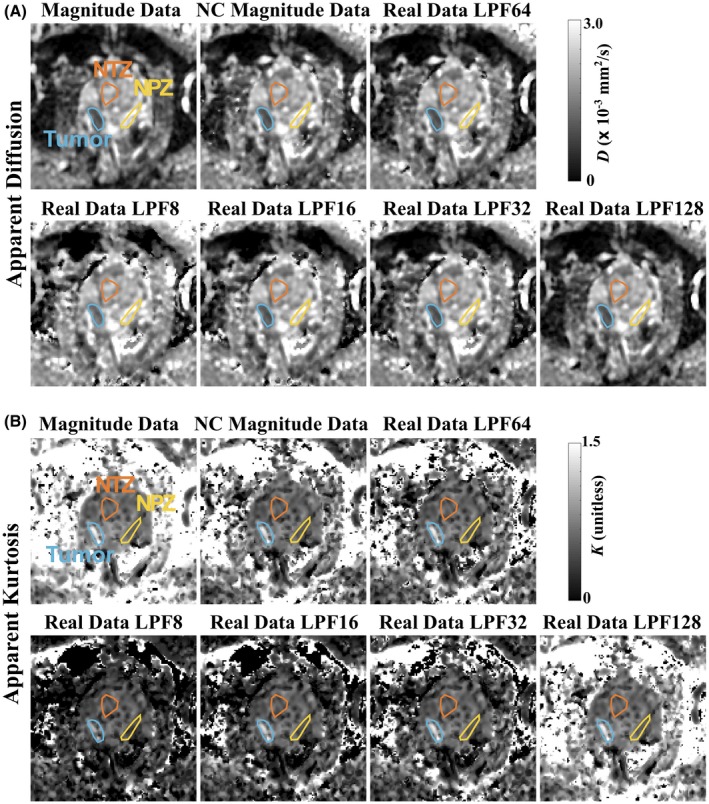
Same‐slice, masked *D* (A) and *K* (B) maps estimated from magnitude data, magnitude data with NC, and real data with phase correction performed with LPF sizes of 8, 16, 32, 64, and 128 pixels, respectively. Zero‐value masking was determined from low‐signal thresholding

Figure 2A compares prostate images where reconstruction was performed with magnitude and real data (phase correction with 64‐pixel LPF). In contrast to *b*‐zero‐acquired images, a clear difference is apparent between the *b* = 1500 s/mm^2^ magnitude‐ and real‐data images, where SNRs are lowest; the mean value of noisy regions can be seen to be higher on the magnitude images than the real images. The intensity‐profile plots below each pair of images confirm this biasing effect in the high‐*b*‐value case and demonstrate how real‐data images can improve contrast for small intensity differences close to the noise floor. Comparing contrast‐to‐noise ratios (CNRs) of ROIs (shown in Figure 3) for these images and their corresponding noise images, magnitude data produced CNRs of 37 for tumor/NPZ, and real data gave 49 for tumor/NPZ.

Figure 2B demonstrates the reduction in noise‐floor levels using real‐data reconstruction by illustrating pixel‐intensity distributions for zero‐SNR noise images reconstructed using magnitude‐ and real‐data averaging. Real‐data histograms are closer to the ideal case with zero‐mean noise statistics, where biases increase with increasing phase‐correction LPF size; in this example, the mean value of the magnitude data is 108.3, compared to 7.4, 15.6, 31.9, 60.1, and 89.9 for real data reconstructed using phase‐correction LPFs of 8, 16, 32, 64, and 128 pixels, respectively. Similar results were seen across all patients, where the value of mean intensity values for noise images in a central 50 × 30‐pixel region ranged from 87.2‐126.2 for magnitude data and 59.6‐69.4 for real data with phase correction performed with 64‐pixel LPF. It is expected that the residual background offsets in real‐valued images are due to the imperfect performance of the method to simultaneously reduce noise‐floor bias and minimize phase‐related artifacts (see the Simulations section).

### 3.4 Apparent diffusion and apparent kurtosis maps

Figure 3 shows *D* and *K* maps estimated for an example patient slice, comparing maps calculated from magnitude data without NC, magnitude data with NC, and real data with phase correction performed with the range of LPF sizes. Also shown are overlaid ROIs for tumor, NPZ, and NTZ. For *D* maps, 8‐pixel‐LPF real data appear to yield the least tumor‐to‐normal tissue contrast. Across the *K* maps, tumor and NPZ are vary visibly, where, compared to magnitude data without NC, mean tumor/NPZ values were found to be 8%/11% lower in maps calculated with NC, and ranged from 28‐3 to 28‐5% lower in *K* maps calculated from real data with phase‐correction LPF sizes from, respectively, 8‐128 for this patient.

### 3.5 Comparison of metrics across patients

**Figure 4:**
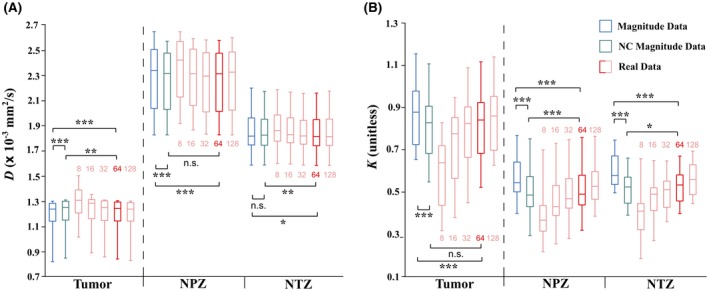
Boxplots showing distributions of prostate DKI metrics in 16 patients estimated using magnitude data without (blue) and with (green) NC, and real data (red) with phase correction performed using LPF sizes of 8, 16, 32, 64, and 128 pixels. Comparisons are made for ROI metrics in tumor, NPZ, and NTZ calculated from *D* (A) and *K* (B) maps. Paired Wilcoxon‐test levels of significance of patient DKI metrics derived from the different methods are indicated as ***P < 0.001, **P < 0.01, *P < 0.05, and n.s. *P* > 0.05

Friedman tests indicated that real data (all LPF sizes) and magnitude data with and without NC yielded *D* and *K* values that were significantly different from each other (*K P* < 0.001 in tumor and normal tissue; *D P* < 0.001 in tumor, *P* < 0.001 in NPZ, *P* < 0.01 in NTZ). The boxplots in Figure 4 illustrate the median, quartile and extreme values (across 16 patients) of *D* and *K* maps calculated with magnitude data without NC, magnitude data with NC, and real data with phase correction performed using the range of tested LPF sizes. Supporting Information Table S1 summarizes these median values and IQRs, as well as percentage differences of the medians and paired Wilcoxon‐test levels of significance with respect to magnitude‐data without NC. Both NC and real data generated significantly (*P* < 0.001) lower *K* metrics than magnitude data without NC in all ROIs. Comparing 2 methods of noise removal, magnitude data with NC produced median *K* 6‐11% lower, while 64‐pixel‐LPF real‐data *K* values were 4‐10% lower. Choice of LPF size appeared to strongly affect *K* metrics: relative to magnitude data without NC, *K* metrics were 25‐33%, 12‐21%, 6‐14%, and 2‐3% lower for LPF sizes of 8, 16, 32, and 128 pixels, respectively.

## 4 DISCUSSION AND CONCLUSIONS

The biophysical modelling technique of DKI can wrongly attribute the nonmonoexponential decay of magnitude data confounded with its noise‐floor to the kurtosis effect, artificially increasing the measured value of K above the value it would have in an idealized noise‐free situation. Indeed, our simulations in this study indicated uncorrected magnitude data can result in a ~7% increase of true K values in NPZ tissue. This is concerning and may account for the mixed results from earlier studies^29^, ^30^, ^31^, ^32^, ^33^, ^34^ that compare DKI with DWI for PCa, where often no noise‐correction method is performed at all. Here, we have investigated a real‐data reconstruction approach to noise bias removal from DKI in the prostate. Our findings showed that DKI metrics derived from such phase‐corrected real data and those fitted using magnitude data with NC were mostly similar (>±2%) and sometimes statistically insignificant.

To reduce spurious increases in K arising from the effect of the rectified noise floor on fitted DKI data, a post hoc NC method of standard magnitude‐averaged images has generally been used.^41^ NC attempts to correct for noise‐floor bias by finding the mean value within a local region of processed noise‐only images, whereas phase‐corrected real data should reduce or eliminate noise floor bias seen in magnitude images, avoiding the need for NC.^36^

In this work, we simulated magnitude‐data and phased‐corrected real‐data reconstructions of a digital phantom based on realistic *D*, *K*, and SNR values. Phase correction was tested with LPFs of 8, 16, 32, 64, and 128‐pixel 2D Fourier‐domain Hamming windows. These tests demonstrated the importance of LPF selection, where lower sizes removed noise biases more successfully but very low‐size LPFs introduced phase‐related artifacts. DKI‐model fitting of the simulated data generated *D* metrics that had a <1% bias in relation to the true values where magnitude data was used (both with and without NC), and real data produced similarly accurate results for LPF sizes of 32 pixels and above. *K* values generated from magnitude data were less accurate (2‐7% biased). Considering all signal models, the 64‐pixel LPF was found to be the most optimal and improved accuracy in both simulated tumor and NPZ tissue compared to the use of NC. This result is supported by our clinical‐data investigations that showed that real‐data *D* and *K* metrics generated using a 64‐pixel LPF were most similar to NC metrics, where NC is a gold‐standard for this application.

As with the simulated results, the lowest (8‐pixel) LPFs generate *K* values that are substantially lower than expected while the highest (128‐pixel) filters produce *K* values slightly higher than expected. This increase of *K* with LPF size from below to above the true value is likely due to the combined effects of incomplete removal of phase‐cancellation artifacts with increasing influence of non‐zero noise biases. Optimal choice of phase‐correction LPF size is, therefore, an important factor in obtaining accurate DKI metrics in prostate in real‐data approaches.

The main advantage that a real‐data approach to noise correction has over NC is that it does not require the acquisition of additional noise images, so that an automated or scanner‐integrated real‐data reconstruction could reduce patient scan times. However, it is difficult to know whether the correct parameters (i.e., LPF size) are being used.

One limitation of this study is that simulated reconstructions did not incorporate parallel‐imaging unaliasing or homodyne detection for multichannel, undersampled, partial‐Fourier data, unlike the manufacturer reconstruction method. Moreover, the optimal value of the LPF may be different for experiments with substantially different signal and noise, e.g., for much higher or lower resolution, different coils or for different tissue types and body regions. While this is not expected to vary greatly, such variations should be tested with simulations in future work. A second limitation is the selection of patients with previous negative biopsy, which may create selection bias, particularly for smaller tumors and in more anterior locations; however, this should not affect the results given the lesion‐based assessment employed.

Our work here and studies using similar approaches in neuro^36^, ^37^, ^43^, ^47^, ^48^ and cardiac imaging^49^ have demonstrated phase‐corrected real (or complex) data reconstruction offers potential advantages in diffusion MRI. Therefore, we will make our adapted reconstruction available on the GE MR Collaboration Community.

In conclusion, real‐data reconstruction may be an alternative to NC for removing or reducing the noise‐floor induced bias in DKI parameters *D* and *K*. However, LPF size has a significant effect on results so should be chosen with care. Results demonstrated with simulations and in patients that a 64‐pixel LPF produces accurate DKI metrics, which are similar to those generated using NC.

## Supporting information


**FIGURE S1** A comparison of clinical phase images across all *b*‐values and diffusion‐encoding directions for an example slice with modeled phase for our simulated diffusion phantom. Dashed yellow lines indicate the position of the prostate
**TABLE S1** Median (IQR) of DKI metrics in prostate ROIs across 16 patients and percentage difference from the median metrics calculated with magnitude‐data without NC. Paired Wilcoxon‐test levels of significance of the patient DKI metrics with comparisons made for magnitude‐data without NC are indicated as ***P < 0.001, **P < 0.01, and *P < 0.05
