# Supplementary material for: Removing rician bias in diffusional kurtosis of the prostate using real‐data reconstruction
Source: Magn Reson Med. 2019 Nov 18;83(6):2243–52. doi: 10.1002/mrm.28080 (PMC7065237; doi:10.1002/mrm.28080)
Supplement: Supplementary file 1 — FIGURE S1 A comparison of clinical phase images across all b‐values and diffusion‐encoding directions for an example slice with modeled phase for our simulated diffusion phantom. Dashed yellow lines indicate the position of the prostate TABLE S1 Median (IQR) of DKI metrics in prostate ROIs across 16 patients and percentage difference from the median metrics calculated with magnitude‐data without NC. Paired Wilcoxon‐test levels of significance of the patient DKI metrics with comparisons made for magnitude‐data without NC are indicated as ***P < 0.001, **P < 0.01, and *P < 0.05 [file MRM-83-2243-s001.docx]

Supporting Information Figure S1: A comparison of clinical phase images across all *b*-values and diffusion-encoding directions for an example slice with modelled phase for our simulated diffusion phantom. Dashed yellow lines indicate the position of the prostate.

Supporting Information Table S1: Median (IQR) of DKI metrics in prostate ROIs across 16 patients and percentage difference from the median metrics calculated with magnitude-data without NC. Paired Wilcoxon-test levels of significance of the patient DKI metrics with comparisons made for magnitude-data without NC are indicated as ****P*<0.001, ***P*<0.01, and **P*<0.05.

| ROI label | **Magnitude data** | **Magnitude data & NC** | | **Real data LPF64** | |
| --- | --- | --- | --- | --- | --- |
| Apparent Diffusion (*D,* x 10^-3^ mm^2^/s) | | | | | |
| Tumor | 1.24 (0.14) | 1.25 (0.15) | ***+1% | 1.24 (0.15) | ***+0.4% |
| NPZ | 2.34 (0.45) | 2.31 (0.43) | ***-1% | 2.31 (0.44) | ***-1% |
| NTZ | 1.81 (0.19) | 1.82 (0.19) | +0.5% | 1.81 (0.19) | *-0.2% |
| Apparent kurtosis (*K,* unitless) | | | | | |
| Tumor | 0.88 (0.24) | 0.83 (0.21) | ***-6% | 0.84 (0.23) | ***-4% |
| NPZ | 0.55 (0.13) | 0.49 (0.13) | ***-11% | 0.49 (0.13) | ***-10% |
| NTZ | 0.68 (0.13) | 0.63 (0.12) | ***-8% | 0.64 (0.11) | ***-7% |

| ROI label | **Real data LPF8** | | **Real data LPF16** | |
| --- | --- | --- | --- | --- |
| Apparent Diffusion (*D,* x 10^-3^ mm^2^/s) | | | | |
| Tumor | 1.31 (0.17) | ***+6% | 1.28 (0.14) | ***+4% |
| NPZ | 2.42 (0.41) | *+4% | 2.31 (0.44) | -1% |
| NTZ | 1.86 (0.20) | +2% | 1.83 (0.19) | +0.6% |
| Apparent kurtosis (*K,* unlitless) | | | | |
| Tumor | 0.64 (0.27) | ***-27% | 0.78 (0.27) | *** -12% |
| NPZ | 0.37 (0.11) | ***-33% | 0.43 (0.12) | ***-21% |
| NTZ | 0.51 (0.11) | ***-25% | 0.59 (0.10) | ***-13% |

| ROI label | **Real data LPF32** | | **Real data LPF128** | |
| --- | --- | --- | --- | --- |
| Apparent Diffusion (*D,* x 10^-3^ mm^2^/s) | | | | |
| Tumor | 1.25 (0.15) | ***+1% | 1.24 (0.14) | <0.1% |
| NPZ | 2.29 (0.45) | **-2% | 2.32 (0.44) | **-1% |
| NTZ | 1.82 (0.18) | +0.1% | 1.81 (0.19) | **-0.2% |
| Apparent kurtosis (*K,* unitless) | | | | |
| Tumor | 0.83 (0.23) | ***-6% | 0.86 (0.24) | ***-2% |
| NPZ | 0.48 (0.13) | ***-14% | 0.53 (0.12) | ***-3% |
| NTZ | 0.61 (0.12) | ***-10% | 0.66 (0.13) | ***-3% |
